# Lentiviral Dendritic Cell Vaccine Targeting Claudin-18.2 Elicits Potent Antitumor Immunity Against Gastric Cancer

**DOI:** 10.3390/cancers18030441

**Published:** 2026-01-29

**Authors:** Bowen Zheng, Wenqing Zhang, Dan Zhou, Miao Fu, Fanzhuoran Lou, Xintian Huang, Xiaowen Xie, Yunli Gong, Kaiyi Rong, Yongxiang Hong, Yanyan Zhan, Li Xiao, Tianhui Hu

**Affiliations:** 1 Xiamen Key Laboratory for Tumor Metastasis, Cancer Research Center, State Key Laboratory of Vaccines for Infectious Diseases, Xiang An Biomedicine Laboratory, Xiamen University, Xiamen 361102, China; 2 Department of Oncology, Zhongshan Hospital of Xiamen University, School of Medicine, Xiamen University, Xiamen 361004, China; 3 Fujian-Taiwan Smart Health and Elderly Care Research Center, Xiamen City University, Xiamen 361008, China; 4 Shenzhen Research Institute of Xiamen University, Shenzhen 518057, China

**Keywords:** gastric cancer, Claudin-18.2 (CLDN18.2), dendritic cell vaccine, immunotherapy, cytotoxic T lymphocytes

## Abstract

**Background:** Claudin-18.2 (CLDN18.2) has emerged as a promising therapeutic target for gastric cancer due to its frequent and specific expression in malignant lesions. Dendritic cell (DC)-based vaccines represent a potent strategy for inducing antitumor immunity; however, their efficacy against solid tumors, such as gastric cancer, remains challenging. **Methods:** We developed a lentiviral vector encoding human CLDN18.2 (Lv-CLDN18.2) to generate antigen-loaded DC vaccines. In vitro, human monocyte-derived DCs were transduced and co-cultured with autologous T cells to induce cytotoxic T lymphocytes (CTLs). CTL function was assessed by flow cytometry, cytokine ELISA, and cytotoxicity assays against CLDN18.2-positive gastric cancer cells. In vivo, the therapeutic efficacy of the DC vaccine was evaluated in a syngeneic mouse model subcutaneously inoculated with MFC-CLDN18.2 cells. **Results:** We successfully produced high-titer Lv-CLDN18.2 and established stable CLDN18.2-positive gastric cancer cell lines. Lv-CLDN18.2-transduced DCs exhibited a mature phenotype with upregulated co-stimulatory (CD80/CD86) and antigen-presenting molecules (HLA-ABC/DR). These DCs potently stimulated CTLs, leading to a significantly higher proportion of activated CD8^+^CD25^+^ T cells, enhanced secretion of IFN-γ and TNF-α, and potent, specific lysis of CLDN18.2-positive target cells in vitro. In mouse models, vaccination with Lv-CLDN18.2-DCs significantly suppressed tumor growth, which was associated with robust CD8^+^ T cell infiltration, reduced tumor cell proliferation (Ki-67), and decreased CLDN18.2-positive tumor cells in vivo. **Conclusions:** Our study demonstrates that a CLDN18.2-targeting DC vaccine can effectively induce potent antigen-specific CTL responses and elicit significant antitumor immunity in a preclinical model. These findings provide a strong rationale for the clinical development of CLDN18.2-directed DC-based immunotherapy for gastric cancer.

## Simple Summary

This study successfully developed a dendritic cell (DC) vaccine targeting CLDN18.2 using a lentiviral vector (Lv-CLDN18.2). The lentivirus efficiently delivered the CLDN18.2 gene into DCs, enabling stable antigen expression and promoting DC maturation. In vitro experiments demonstrated that the vaccine activated specific CD8^+^ T cells, which effectively lysed CLDN18.2-positive gastric cancer cells. In a syngeneic mouse model, vaccination significantly suppressed tumor growth and was associated with increased intratumoral infiltration of CD8^+^ T cells. These results indicate that the lentivirus-based CLDN18.2-DC vaccine represents a promising immunotherapeutic strategy for treating gastric cancer.

## 1. Introduction

Gastric cancer (GC) represents a major global health burden, being the fifth most prevalent malignancy and the fourth leading cause of cancer-associated deaths worldwide [1]. In cases of locally advanced or metastatic disease, conventional therapeutic approaches primarily involve chemotherapy, trastuzumab for HER2-positive tumors, and, more recently, immune checkpoint blockade therapies (ICIs) [2]. Although ICIs such as PD-1 inhibitors benefit some patient subgroups, overall response rates in gastric cancer remain generally low, underscoring the necessity to turn “cold” tumors “hot” immunologically [3]. The prognosis for advanced disease is poor, with 5-year survival rates of only 5–20% [4], due to factors like delayed detection, aggressive tumor characteristics, and acquired treatment resistance. Consequently, there is a pressing need for innovative treatment and well-tolerated therapies that can significantly extend patient survival.

An optimal therapeutic target for cancer is characterized by broad expression on tumor cells and minimal presence in healthy tissues, minimizing unintended toxic effects. Claudin-18.2 (CLDN18.2), a gastric-specific tight junction protein, meets this criterion, making it a transformative target in gastric cancer (GC) [5]. Under normal physiological conditions, CLDN18.2 is confined to the gastric mucosa and is largely inaccessible to immune surveillance. Malignant transformation disrupts tight junctions, leading to widespread CLDN18.2 redistribution over the entire cell surface and thereby rendering it a suitable target for immunotherapy [6]. In the SPOTLIGHT and GLOW trials, CLDN18.2 expression was detected in approximately 70% of gastric cancer (GC) cases, with 38% exhibiting moderate-to-strong (2+/3+) membranous staining using the VENTANA CLDN18 (43-14A) RxDx Assay [7,8]. This expression profile underscores its potential as a valuable therapeutic target. Recently, the clinical efficacy of CLDN18.2-targeted therapy was demonstrated by zolbetuximab, a monoclonal antibody that, in combination with chemotherapy, significantly improved survival outcomes in patients with CLDN18.2-postive gastric cancer, representing a notable advance in precision medicine [9]. These encouraging results have spurred further development of alternative CLDN18.2-targeting strategies, including bispecific antibodies, antibody–drug conjugates (ADCs), and engineered cellular immunotherapies [10,11].

In addition to passive immunotherapies, active immunization strategies that stimulate the patient’s own immune system to generate sustained antitumor responses show significant potential for establishing long-term immunological memory. Among these, dendritic cell (DC)-based vaccines represent a prominent approach [12]. As highly specialized antigen-presenting cells, DCs are critical for capturing, processing, and presenting tumor-associated antigens to naïve T cells, thereby initiating and directing adaptive immune responses [13]. The rationale for DC vaccination involves isolating patient-derived DCs, loading them ex vivo with tumor antigens, and reinfusing them to induce a potent, polyclonal, and tumor-specific T-cell response [14]. However, the clinical efficacy of DC vaccines in solid tumors has been variable, often constrained by suboptimal antigen presentation, inadequate DC activation leading to T-cell tolerance, and the immunosuppressive tumor microenvironment [15,16].

We hypothesized that combing the high-selectivity CLDN18.2 biomarker with an advanced dendritic cell vaccination strategy could overcome current therapeutic limitations and induce a potent antitumor response. Conventional DC vaccine methods have largely relied on short peptide loading or crude tumor lysates; in contrast, our approach utilizes lentiviral vector-mediated antigen delivery. This system offers distinct advantages: it ensures effective cellular uptake and sustained endogenous production of the full-length target protein, facilitating antigen processing through both MHC class I and II pathways to activate cytotoxic CD8^+^ and helper CD4^+^ T-cell populations [17]. Furthermore, the lentiviral delivery system itself possesses intrinsic immunostimulatory properties—likely mediated through microbial pattern recognition receptors—that function as a built-in adjuvant to promote DC maturation, enhance surface costimulatory molecule expression, and improve T-cell priming capacity [18]. This genetically engineered antigen-presenting cell platform has shown promising efficacy in preclinical models across multiple tumor types [19].

In this study, we developed and evaluated a novel dendritic cell (DC) vaccine engineered via lentiviral vectors to express CLDN18.2 for the treatment of gastric cancer. We systematically assessed the vaccine’s ability to elicit CLDN18.2-specific cytotoxic T lymphocyte responses in vitro and evaluated its therapeutic efficacy along with corresponding immune correlates in a syngeneic mouse model in vivo. This work aims to establish a rational scientific basis for a new active immunotherapeutic strategy against CLDN18.2-positive gastric cancer, with potential implications for future clinical development.

## 2. Materials and Methods

### 2.1. Patient Eligibility

Peripheral blood samples were obtained from healthy donors (aged 18–65 years) at Zhongshan Hospital, Xiamen University. All participants provided written informed consent prior to blood collection. The study was approved by the Medical Ethics Committee of Zhongshan Hospital, Xiamen University (Approval No. xmzsyyjy-2024-151; approval date: 13 May 2024).

### 2.2. Cell Lines and Culture Conditions

Human gastric adenocarcinoma AGS (CLDN18.2-negative), HGC27 (CLDN18.2-negative), and KATO III (CLDN18.2-positive) cells, mouse forestomach carcinoma MFC cells, and human embryonic kidney HEK293T cells were obtained from the Shanghai Cell Bank (Shanghai, China). All cell lines were authenticated by short tandem repeat (STR) profiling and confirmed to be mycoplasma-free. HEK293T cells were maintained in DMEM (Gibco, 12800017) (Waltham, MA, USA); AGS, HGC27, and MFC cells were maintained in RPMI-1640 (Gibco, 31800022). All media were supplemented with 10% fetal bovine serum (FBS; Gibco, 10270106), 100 U mL^−1^ penicillin, and 100 µg mL^−1^ streptomycin (Life Technologies, Carlsbad, CA, USA). Cells were incubated at 37 °C in a humidified 5% CO_2_ atmosphere and passaged every 3–4 days.

### 2.3. Plasmid Construction and Lentivirus Production

Human CLDN18.2 cDNA was amplified from KATO III-derived cDNA; murine Cldn18.2 cDNA was amplified from normal mouse stomach tissue. Both fragments were PCR-flag-tagged and sub-cloned into the pLVX lentiviral backbone (Addgene, 85140) (Watertown, MA, USA). Constructs were verified by Sanger sequencing. For virus production, 2.5 × 10^7^ HEK293T cells were seeded per 15 cm dish. Cells were transfected with pLVX-CLDN18.2 (or empty vector), pMDL (Addgene, 12253), pVSVG and pRSV-Rev using PEI transfection reagent (Yeasen, 40816ES01) (Shanghai, China). Supernatants were harvested 72 h later, cleared (500 *g*, 10 min, 4 °C), filtered (0.45 µm PVDF; Millipore, HAWP04700) (Burlington, MA, USA) and precipitated with 1/4 volume ice-cold 5× PEG-6000/NaCl overnight at 4 °C. Particles were pelleted (7000 *g*, 10 min, 4 °C), resuspended in ice-cold PBS, aliquoted and stored at −80 °C. Lentiviral titer was determined with the TransLy™ Lentivirus qPCR Titration Kit (TransGen, FV201) (Beijing, China).

### 2.4. Lentivirus Characterization by Electron Microscopy and Nanoparticle Tracking

Concentrated lentivirus (10 µL) was adsorbed onto 200-mesh carbon-coated copper grids, negatively stained with 1% uranyl acetate for 15 s, and air-dried for 3 h. Samples were examined with an H-7650 transmission electron microscope (Hitachi, Tokyo, Japan) at 80 kV. Size distribution was further analyzed by nanoparticle tracking analysis (NTA) on a NanoSight NS300 (Malvern Panalytical, Worcestershire, UK). Each sample was measured in triplicate.

### 2.5. Transduction and Stable Cell Line Generation

Target cells seeded at 40–50% confluence in 6-well plates were transduced with concentrated lentivirus plus 8 µg mL^−1^ polybrene (Yeasen, 40804ES76). After 48 h, stable pools were selected with 2 µg mL^−1^ puromycin.

### 2.6. RNA Extraction, Reverse Transcription and qPCR

Total RNA was isolated using TRIzol reagent (TaKaRa, 9109) (Kusatsu, Shiga, Japan). One microgram of RNA was reverse-transcribed with a PrimeScript RT Reagent Kit (TaKaRa, RR036A). qPCR was performed on a LightCycler 96 instrument (Roche, Basel, Switzerland) using TB Green Premix Ex Taq II (TaKaRa, RR820). Relative expression was calculated by the 2^−ΔΔCt^ method with GAPDH as endogenous control. Primer sequences are listed in Supplementary Table S1.

### 2.7. Western Blotting

Cells were lysed in RIPA buffer containing protease inhibitors (Thermo Fisher, 693132001) (Waltham, MA, USA). Protein concentration was determined with a BCA kit (Thermo Fisher, 23227). Equal amounts were resolved by SDS-PAGE, transferred to PVDF membranes and blocked with 5% skimmed milk. Membranes were incubated overnight at 4 °C with primary antibodies (all 1:1000): CD8α (Immunoway, YM8067) (San Jose, CA, USA), CLDN18.2 (Immunoway, YM9290), Ki-67 (Immunoway, YM8189), β-tubulin (Immunoway, YM8332), and HRP-conjugated goat anti-rabbit IgG (Immunoway, RS0002) were applied at 1:100,000. Signals were visualized with ECL reagent (Abbkine Scientific Co., Ltd., BMU102) (Wuhan, China) on a Gel Doc XR System (Bio-Rad, Hercules, CA, USA).

### 2.8. ELISA Detection of TNF-α and IFN-γ

To measure levels of TNF-α and IFN-γ in cell supernatants and mouse tumor lysates, utilize specific ELISA kits (human TNF-α: RK05051; mouse TNF-α: RK04595; human IFN-γ: RK05052; mouse IFN-γ: RK00019) (Abclonal, Wuhan, China) following the manufacturer’s instructions. Briefly, equilibrate reagents to room temperature, prepare samples, add to microtiter plates, incubate, wash, add detection reagents, and measure optical density at 450 nm using a microplate reader. Calculate cytokine concentrations using the standard curve provided with each kit.

### 2.9. Flow Cytometry

For the phenotypic analysis of human dendritic cells (DCs) and T cells, single-cell suspensions were prepared and stained. Briefly, cells were resuspended in flow cytometry staining buffer and incubated with fluorochrome-conjugated antibodies for 30 min on ice in the dark. After washing, cells were fixed, and data were immediately acquired on a BD FACSCanto II flow cytometer. For apoptosis detection, cells were stained with Annexin V-FITC and propidium iodide (PI) according to the manufacturer’s instructions. The following antibodies were used: APC anti-human CD14 (BioLegend, 301806) (San Diego, CA, USA), FITC anti-human CD11c (BioLegend, 301604), PE anti-human HLA-DR (BioLegend, 317416), APC anti-human HLA-ABC (Thermo Fisher, 17-9983-42), ABflo™ 647 anti-human CD80 (Abclonal, A23730) (Wuhan, China), PE anti-human CD86 (Thermo Fisher, 12-0869-42), Brilliant Violet 650 anti-human CD3 (Thermo Fisher, 16-0038-42), APC anti-human CD4 (BioLegend, 301006), FITC anti-human CD8a (BioLegend, 307606), and PE anti-human CD25 (Thermo Fisher, 12-0259-42). Flow cytometry data were acquired using a BD FACSCanto II and analyzed with FlowJo software (version 10).

### 2.10. Generation of Human Dendritic Cell Vaccines

Peripheral blood (50 mL) was collected from healthy donors under informed consent. PBMCs were isolated with the Human Peripheral Blood Monocyte Isolation Solution Kit (Solarbio, P8680) (Beijing, China). Adherent monocytes (5 × 10^6^ per well) were cultured in RPMI-1640 + 10% FBS containing 100 ng mL^−1^ recombinant human GM-CSF (Abclonal, RP00094) and 50 ng mL^−1^ IL-4 (Abclonal, RP01703). Half-medium changes were performed every 48 h. On day 5, immature DCs (iDCs) were harvested and infected with Lv-CLDN18.2 or Lv-Vec at MOI = 200 plus 8 µg mL^−1^ polybrene. After 24 h, 10 ng mL^−1^ recombinant human TNF-α (Abclonal, RP00993) was added. Mature DCs were collected on day 10 and analyzed for CD11c, HLA-ABC, HLA-DR, CD80 and CD86 expression by flow cytometry.

### 2.11. T-Cell Isolation and Expansion

Non-adherent autologous PBMCs were resuspended at 2–5 × 10^6^ cells mL^−1^ in complete RPMI-1640. CD3^+^ T cells were positively selected with CD3/CD28-conjugated magnetic beads (Abclonal, A25789) at a 3:1 bead/cell ratio and stimulated with 500 IU mL^−1^ recombinant human IL-2 (Thermo Fisher, RP01039). After 48 h, cells were maintained in 100 IU mL^−1^ IL-2 for an additional 5–7 days.

### 2.12. In Vitro CTL Induction and Cytotoxicity Assay

Mature DCs were co-cultured with autologous T cells at a 1:10 ratio in complete medium supplemented with 100 IU mL^−1^ IL-2. After 24 h, CTLs were harvested and stained for CD4, CD8, and CD25.

For cytotoxicity, AGS~CLDN18.2 or HGC27~CLDN18.2 target cells were seeded at 5 × 10^3^ cells per well in 96-well plates. CTLs were added at the indicated effector/target (E:T) ratios. After 24 h, viability was measured using the CCK-8 reagent (APExBIO, K1018) (Houston, TX, USA). Percent inhibition was calculated as

Cytotoxicity (%) = [1 − (ODexperimental − ODblank)/(ODtarget alone − ODblank)] × 100

### 2.13. Generation of Murine Bone-Marrow-Derived DC Vaccines

Bone marrow was flushed from the femurs and tibiae of 6–8-week-old male 615-line mice. Red blood cells were lysed, and the remaining cells were washed twice. Cells were plated at 2 × 10^5^ mL^−1^ in 10 cm bacterial-grade dishes in 10 mL RPMI-1640 + 10% FBS containing 20 ng mL^−1^ murine GM-CSF (Abclonal, RP01206) and 10 ng mL^−1^ murine IL-4 (Abclonal, RP01161). On day 3, an additional 10 mL of cytokine-containing medium was added. On day 6, non-adherent and loosely adherent iDCs were harvested, re-plated in tissue-culture-grade dishes, and infected with Lv-CLDN18.2 or Lv-Vec (MOI = 200) plus 8 µg mL^−1^ polybrene. After 24 h, 10 ng mL^−1^ murine TNF-α (Abclonal, RP01071) was added. Mature BMDCs were collected on day 10, and antigen loading was verified by Western blot.

### 2.14. In Vivo Tumor Challenge and Treatment

Twenty male 6–8-week-old 615-line mice (catalog number: M0026) were housed under SPF conditions; all procedures were approved by Xiamen University IACUC (XMULAC20240239). Inclusion criterion: develop a palpable tumor within 7 days of s.c. inoculation of 1 × 10^6^ MFC-CLDN18.2 cells in the right flank. Five mice that failed to form tumors were excluded a priori, leaving 16 eligible animals. Power analysis (pilot data: Δ = 250 mm^3^, SD = 60 mm^3^, 80% power, α = 0.05) indicated n = 3.5 per group; we used n = 4 and randomized the 20 mice into four equal groups (untreated, PBS-DC, Lv-Vec-DC, Lv-CLDN18.2-DC) by Excel RAND with allocation concealment. DC vaccines (1 × 10^6^ cells per mouse) were given i.v. on days 7, 14 and 21. Tumor size (calipers every 2 days, volume = 0.5 × length × width^2^) and body weight were recorded by a blinded investigator. Humane endpoints: tumor length ≥ 15 mm, ulceration, weight loss ≥ 20%, or distress; mice reaching any limit were immediately euthanized by CO_2_ plus cervical dislocation. On day 28, 150 mg kg^−1^ D-luciferin was injected i.p. and bioluminescence images (IVIS Spectrum) were acquired at 10 min; tumors were then excised, weighed and fixed in 4% paraformaldehyde for histology.

### 2.15. Histology and Immunohistochemistry

Tumors were fixed in 4% paraformaldehyde for 48 h, paraffin-embedded, and sectioned at 4 µm. After antigen retrieval and blocking, sections were incubated overnight at 4 °C with primary antibodies against CLDN18.2 (Abcam, ab222512, 1:200) (Cambridge, UK), CD8α (Proteintech, 66868-1-Ig, 1:200) (Wuhan, China), or Ki-67 (Abcam, ab16667, 1:300) and cleaved caspase 3 (Proteintech, 25128-1-AP, 1:200), followed by HRP-conjugated secondary antibody using the UltraSensitive SP IHC Kit (MXB Biotechnologies, KIT-9710) (Fuzhou, China) and DAB visualization. Positive area percentage was quantified by the ImageJ IHC Profiler plugin (version 2014, SourceForge, NIH, Bethesda, MD, USA) under identical exposure settings (three fields per tumor, n = 4 tumors per group).

### 2.16. Statistical Analysis

Data are presented as mean ± SD. Comparisons among multiple groups were performed with one-way ANOVA followed by Tukey’s multiple-comparison test using GraphPad Prism 9.0. *p* < 0.05 was considered statistically significant.

## 3. Results

### 3.1. Construction and Characterization of CLDN18.2-Expressing Lentiviral Vectors

**Figure 1:**
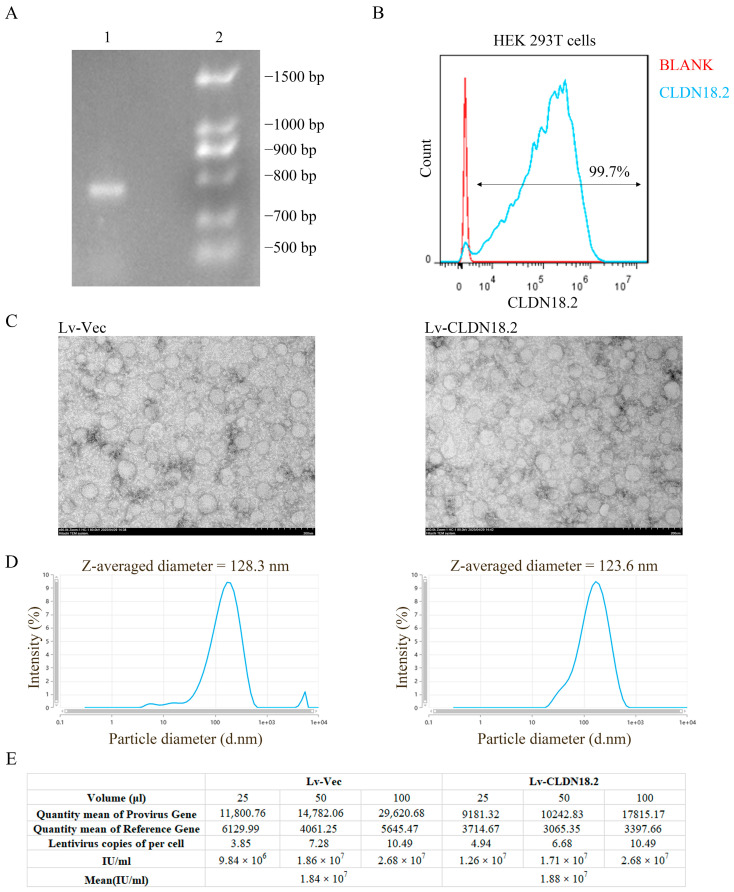
Construction and characterization of the lentiviral vector encoding CLDN18.2. (**A**) Agarose gel electrophoresis of PCR products confirming the successful insertion of the human CLDN18.2 (786 bp) cDNA into the pLVX backbone. Lane 1: pLVX-CLDN18.2; Lane 2: DNA marker. For the original blots, see Supplementary Materials. (**B**) Flow cytometry analysis demonstrating high-efficiency transient expression of CLDN18.2 in HEK293T cells 72 h post-transfection with the pLVX-CLDN18.2 plasmid. (**C**) Representative transmission electron microscopy images of concentrated Lv-Vec (left) and Lv-CLDN18.2 (right) viral particles. Scale bar, 200 nm. (**D**) Nanoparticle tracking analysis (NTA) showing the size distribution of purified Lv-Vec and Lv-CLDN18.2 particles. (**E**) Functional titers of concentrated Lv-Vec and Lv-CLDN18.2, as determined by qPCR. Data are presented as mean ± SD (n = 3 independent preparations).

A lentiviral vector encoding human CLDN18.2 was constructed to generate a potent immunogen. The recombinant plasmid, designated pLVX-CLDX18.2-Puro, was generated by inserting the full-length human *CLDN18.2* cDNA (786 bp) into the vector’s multiple cloning site. Successful insertion was confirmed by PCR and agarose gel electrophoresis (Figure 1A). Sanger sequencing further verified the fidelity of the cloned sequence. To evaluate its eukaryotic expression capacity, the plasmid was transiently transfected into HEK293T cells. Flow cytometry performed 72 h post-transfection demonstrated high-level expression, with approximately 99.7% of cells positive for CLDN18.2 (Figure 1B), confirming the construct’s robust functionality.

The packaged lentiviruses (Lv-Vec and Lv-CLDN18.2) were concentrated and characterized. Transmission electron microscopy images of both preparations revealed scattered, approximately spherical viral particles (Figure 1C). Nanoparticle tracking analysis showed a similar size distribution for both viruses, with a peak diameter around 120 nm (Figure 1D). Functional titrations by quantitative PCR (qPCR) yielded comparable titers of 1.84 × 10^7^ IU/mL for Lv-Vec and 1.86 × 10^7^ IU/mL for Lv-CLDN18.2 (Figure 1E), confirming successful and efficient production of both viral stocks.

### 3.2. Generation of Stable CLDN18.2-Positive Gastric Cancer Cell Lines

To establish target cells for subsequent in vitro and in vivo studies, we transduced the CLDN18.2-negative human gastric cancer cell lines AGS and HGC27, as well as the mouse forestomach carcinoma cell line MFC, with Lv-CLDN18.2 at a multiplicity of infection (MOI) of 50. Following selection with puromycin, Western blot analysis confirmed the successful and stable expression of CLDN18.2 protein in all three transduced cell lines (Supplementary Figure S1).

### 3.3. Generation and Phenotypic Validation of CLDN18.2-Loaded Human Dendritic Cell Vaccines

**Figure 2:**
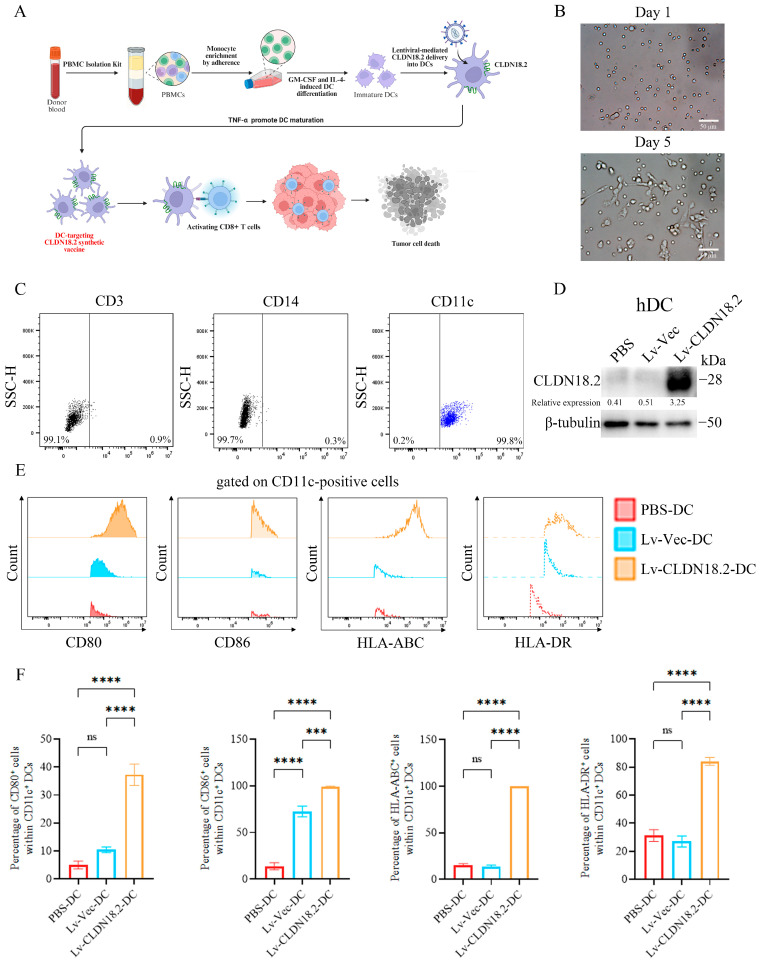
Generation and phenotypic validation of CLDN18.2-loaded human dendritic cell vaccines. (**A**) Schematic diagram of the workflow for generating the DC vaccine and inducing cytotoxic T lymphocytes (CTLs) in vitro. (**B**) Bright-field images showing the morphological differentiation of human monocytes into immature DCs (iDCs) from day 1 to day 5. Scale bar, 50 µm. (**C**) Flow cytometric analysis confirming the high purity of iDCs (CD11c^+^) and absence of monocyte (CD14) and T-cell (CD3) markers. (**D**) Western blot analysis confirming CLDN18.2 protein expression in DCs transduced with Lv-CLDN18.2, but not in PBS-treated or Lv-Vec-transduced controls. β-tubulin served as a loading control. For the original blots, see Supplementary Materials. (**E**,**F**) Flow cytometric analysis of maturation markers on CD11c^+^ gated DCs. Histograms (left) and quantified data (right) demonstrate the upregulated expression of HLA-ABC, HLA-DR, CD80, and CD86 in Lv-CLDN18.2-DCs compared to Lv-Vec-DCs. Data are presented as mean ± SD and are representative of three independent experiments. *** *p* < 0.001, **** *p* < 0.0001, ns > 0.05.

A schematic outlining the preparation of the dendritic cell (DC) vaccine and the subsequent induction of T cell responses is shown in Figure 2A. Human monocytes isolated from PBMCs were differentiated into immature dendritic cells (iDCs) by culturing for 6 days in medium supplemented with GM-CSF and IL-4. Microscopic observation revealed a characteristic morphological progression: from small, round cells on day 1 to large, irregular, and adherent cells with a translucent appearance by day 5, consistent with typical iDC morphology (Figure 2B). The purity of the iDC population was assessed by flow cytometry. The analysis confirmed that 99.8% of the cells expressed the DC marker CD11c, while being negative for the monocyte marker CD14 and the T cell marker CD3 (Figure 2C).

These iDCs were subsequently transduced with Lv-CLDN18.2 or the control Lv-Vec at an MOI of 200 to generate the therapeutic (DC-CLDN18.2) and control (DC-Vec) vaccines, respectively. Specific expression of the CLDN18.2 protein in DC-CLDN18.2 was confirmed by Western blot analysis (Figure 2D). Following maturation induced by TNF-α, the phenotype of the DCs was thoroughly assessed. Flow cytometric analysis, gating on CD11c^+^ cells, demonstrated that DC-CLDN18.2 exhibited a markedly enhanced maturation profile compared to DC-Vec. This was evidenced by significantly higher surface expression of the co-stimulatory molecules CD80 (37.30 ± 3.82% vs. 10.43 ± 0.84%) and CD86 (98.87 ± 1.02% vs. 72.63 ± 5.77%), as well as the antigen-presenting molecules HLA-ABC (99.93 ± 0.06% vs. 13.83 ± 1.46%) and HLA-DR (84.10 ± 2.65% vs. 26.97 ± 4.08%) (Figure 2E,F). These results confirm the successful generation of functionally mature dendritic cells specifically loaded with the CLDN18.2 antigen.

### 3.4. CLDN18.2-DC Vaccines Potently Induce Antigen-Specific Cytotoxic T Lymphocyte (CTL) Responses In Vitro

**Figure 3:**
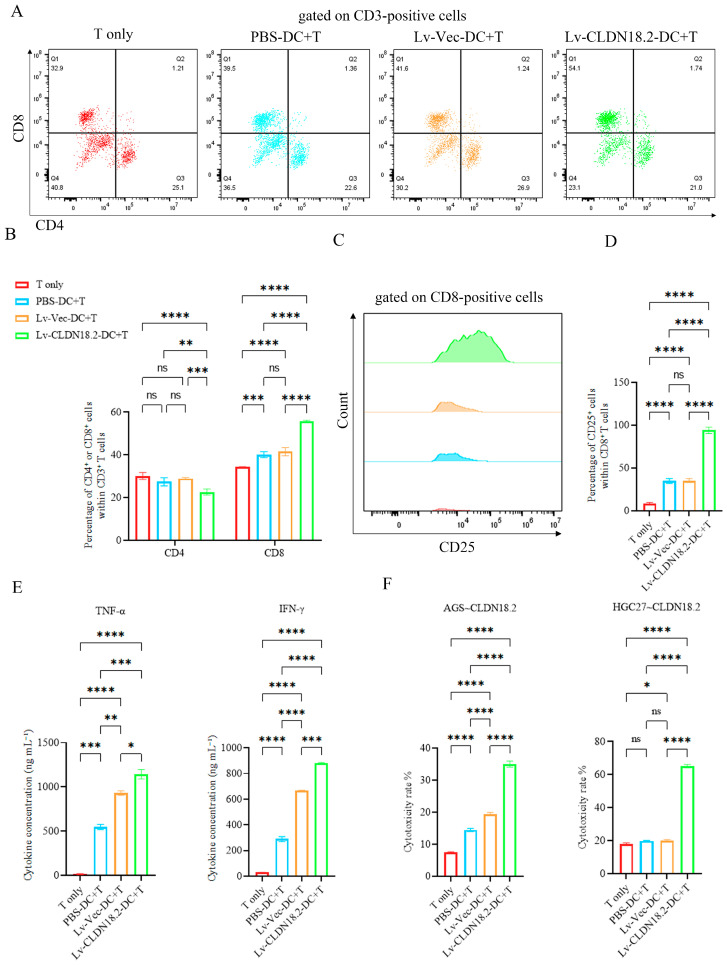
CLDN18.2-DC vaccines induce potent antigen-specific cytotoxic T lymphocyte responses in vitro. (**A**) Representative flow cytometry plots gated on CD3^+^ T cells, showing the proportion of CD4^+^ and CD8^+^ T cells. (**B**) Quantitative analysis of CD4^+^ and CD8^+^ T cells among CD3^+^ cells. Data are presented as mean ± SD (n = 3). (**C**,**D**) Quantitative analysis of CD25 expression on CD8^+^ T cells. Data are presented as mean ± SD (n = 3). (**E**) Cytokine levels of IFN-γ and TNF-α in co-culture supernatants, measured by ELISA. Data are presented as mean ± SD (n = 3). (**F**) In vitro cytotoxicity assay. Specific lysis of AGS~CLDN18.2 and HGC27~CLDN18.2 target cells by CTLs at an effector-to-target (E:T) ratio of 40:1. Data are presented as mean ± SD (n = 5). ns > 0.05, * *p* < 0.05, ** *p* < 0.01, *** *p* < 0.001, **** *p* < 0.0001.

To evaluate the ability of the DC vaccines to stimulate T cells, autologous CD3^+^ T cells were co-cultured with the different DC preparations (T only, PBS-DC, Lv-Vec-DC, or Lv-CLDN18.2-DC) at a 1:10 ratio. After 24 h, the induced CTLs were analyzed. Flow cytometric analysis gated on CD3^+^ cells showed that CTLs induced by Lv-CLDN18.2-DCs contained a significantly higher proportion of CD8^+^ T cells compared to those induced by Lv-Vec-DC (55.80 ± 0.65% vs. 41.53 ± 1.93%; Figure 3A,B). Furthermore, the activation marker CD25 was expressed on a substantially larger fraction of T cells in the Lv-CLDN18.2-DC group (96.13 ± 3.23% vs. 35.27 ± 2.19%; Figure 3C,D). Consistent with this enhanced T cell activation, ELISA of the culture supernatants demonstrated that CTLs from the Lv-CLDN18.2-DC group secreted significantly higher levels of the key effector cytokines IFN-γ (880.83 ± 4.17 pg/mL vs. 668.97 ± 0.45 pg/mL) and TNF-α (1180.57 ± 53.07 pg/mL vs. 931.02 ± 26.15 pg/mL) (Figure 3E).

Most importantly, these primed CTLs exhibited potent and antigen-specific cytotoxic activity in vitro. Using AGS~CLDN18.2 and HGC27~CLDN18.2 cells as target cells, Lv-CLDN18.2-DC-primed CTLs demonstrated the highest killing efficiency at an E:T ratio of 40:1 (Figure 3F). At this ratio, the specific lysis of AGS~CLDN18.2 cells reached 35.05 ± 0.95% for the lv-CLDN18.2-DC group compared to 19.42 ± 0.55% for the Lv-Vec-DC control group. Similarly, specific lysis of HGC27~CLDN18.2 cells was 65.17 ± 1.04% versus 19.87 ± 0.76%, respectively. Apoptosis of target cells was further quantified by flow cytometry. Compared to the control, Lv-CLDN18.2-DC-primed CTLs significantly increased the apoptotic cell percentage in AGS~CLDN18.2 target cells (from 4.67% to 16.00%), and in HGC27~CLDN18.2 target cells (from 11.00% to 24.67%). Notably, the higher baseline CLDN18.2 expression in HGC27~CLDN18.2 cells correlated with a greater proportion of apoptotic cells, supporting both the efficacy and the antigen specificity of the CTL response (Supplementary Figure S2A,B). Conversely, the CTLs showed minimal cytotoxic activity against CLDN18.2-negative parental cell lines, confirming target specificity (Supplementary Figure S2C). Collectively, these data demonstrate that CLDN18.2-loaded DCs prime CTLs that effectively and selectively lyse CLDN18.2-positive tumor cells.

### 3.5. CLDN18.2-DC Vaccines Suppress Tumor Growth and Modulate the Tumor Microenvironment In Vivo

**Figure 4:**
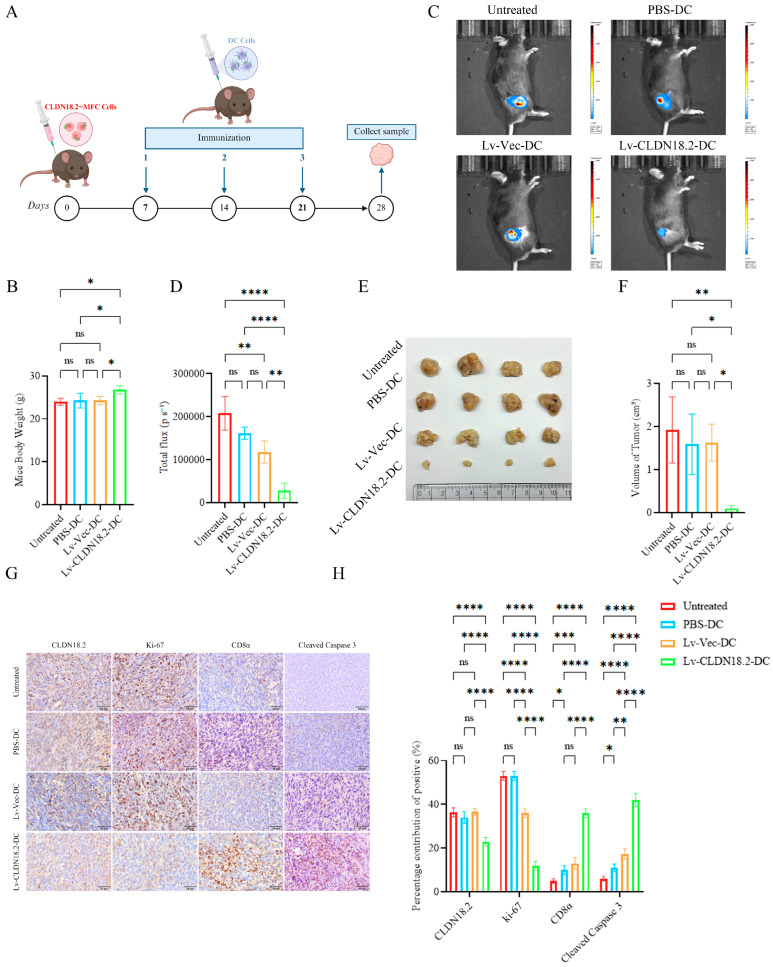
CLDN18.2-DC vaccine suppresses tumor growth and modulates the tumor microenvironment in vivo. (**A**) Experimental timeline of tumor challenge and therapeutic vaccination. (**B**) Body weight curves of mice in different treatment groups. Data are presented as mean ± SD (n = 4 mice per group). (**C**) Representative in vivo bioluminescence images of mice from each group on day 28. (**D**) Quantification of total flux from bioluminescence imaging. Data are presented as mean ± SD (n = 4). (**E**,**F**) Final tumor volumes at the experimental endpoint (day 28). Data are presented as mean ± SD (n = 4). (**G**) Representative immunohistochemistry (IHC) images of tumor sections stained for CLDN18.2, Ki-67, CD8α, and cleaved caspase 3. Scale bar, 50 µm. (**H**) Quantitative analysis of IHC staining. Graphs show the positive area for CLDN18.2, Ki-67, CD8α and cleaved Caspase 3 per field. Data are presented as mean ± SD (n = 4 tumors/group; 3 fields/tumor). ns > 0.05, * *p* < 0.05, ** *p* < 0.01, *** *p* < 0.001, **** *p* < 0.0001.

The therapeutic efficacy of the DC vaccine was evaluated in a syngeneic mouse model. Overall, 615 mice were inoculated subcutaneously with MFC-CLDN18.2 cells and treated with three weekly intravenous doses of the respective DC vaccines once tumors were palpable (Figure 4A). The murine DC vaccines were generated from bone marrow-derived DCs (BMDCs), and successful loading of the CLDN18.2 antigen was confirmed by Western blotting (Supplementary Figure S3). The treatment was well-tolerated, as evidenced by comparable body weights across all groups (Figure 4B).

In vivo bioluminescence imaging on day 28 showed that the Lv-CLDN18.2-DC group had the strongest suppression of tumor growth (Figure 4C,D). This was corroborated by direct measurements of tumor volume (Figure 4E,F), which was significantly lower in the Lv-CLDN18.2-DC treatment group.

Immunohistochemical analysis of the harvested tumors provided mechanistic insights (Figure 4G). Quantification of staining (Figure 4H) demonstrated that tumors from the Lv-CLDN18.2-DC group contained a significantly lower percentage of CLDN18.2-positive cells (23.00 ± 2.00%) compared to the control group (Lv-Vec-DC: 36.67 ± 1.53%; PBS-DC: 34.00 ± 2.65%; untreated: 36.33 ± 2.05%), indicating effective elimination of the antigen-expressing tumor cells. Furthermore, Ki-67 staining confirmed that proliferation was most dramatically reduced in the Lv-CLDN18.2-DC group (12.00 ± 2.01%), significantly lower than in all control groups (Lv-Vec-DC: 36.00 ± 1.86%; PBS-DC: 52.00 ± 2.01%; untreated: 53.00 ± 1.98%). Conversely, cleaved caspase 3 staining indicated a significant increase in apoptosis within the Lv-CLDN18.2-DC-treated tumors (42 ± 2.8%) compared to controls (Lv-Vec-DC: 17 ± 2.1%, PBS-DC: 11 ± 2.2%, untreated: 6 ± 1.5%), supporting the induction of programmed cell death as a key mechanism of action.

Critically, tumors from the Lv-CLDN18.2-DC group exhibited a pronounced increase in CD8^+^ T-cell infiltration (36.00 ± 2.00%), in stark contrast to controls (Lv-Vec-DC: 13.00 ± 2.65%; PBS-DC: 10.00 ± 2.65%; untreated: 5.00 ± 1.00%). And the activation marker CD25 was expressed on a much larger fraction of these T cells in the Lv-CLDN18.2-DC group (90.07 ± 4.23% vs. 28.00 ± 3.19%) (Supplementary Figure S4A,B). Correspondingly, ELISA of tumor lysates showed significantly elevated levels of the effector cytokines IFN-γ (1812.03 ± 51.59 pg/mL vs. 987.9 ± 50.18 pg/mL) and TNF-α (1715.01 ± 54.07 pg/mL vs. 1097.02 ± 46.15 pg/mL) in the Lv-CLDN18.2-DC group (Supplementary Figure S4C). Taken together, these data demonstrate that the CLDN18.2-DC vaccine effectively inhibits tumor growth in vivo, mechanistically linked to enhanced CD8^+^ T cell infiltration within the tumor microenvironment, coupled with reduced tumor cell proliferation and increased apoptosis.

## 4. Discussion

This study developed and comprehensively evaluated a novel dendritic cell (DC) vaccine engineered to target CLDN18.2 for the treatment of gastric cancer. Our experimental data demonstrate the vaccine’s remarkable effectiveness in both laboratory and animal studies. We showed that lentiviral vector delivery serves as an excellent method for generating antigen-presenting DCs while simultaneously promoting their maturation into immunologically active cells. This process ultimately triggers a powerful cytotoxic T-cell reaction against CLDN18.2, showing significant potential for inhibiting tumor progression.

DCs are probably the most potent antigen-presenting cells for CTL induction. DC- based vaccines have been extensively investigated for both prophylactic and therapeutic vaccines against various infections, cancers and other diseases [20]. A major challenge in DC vaccine design is achieving efficient antigen delivery that sustains prolonged MHC-restricted presentation and provides adequate co-stimulatory signals. While peptide-loading strategies are commonly employed for their simplicity, they often result in transient antigen presentation, limited T-cell receptor (TCR) diversity, and a lack of intrinsic “danger” signals required for optimal DC activation [21]. Our lentiviral-based approach effectively addresses these limitations. Its high transduction efficiency, evidenced by near-universal CLDN18.2 protein expression, ensured broad antigen coverage within the DC population. Notably, lentivirally transduced DCs displayed a substantially enhanced maturation characteristic. This aligns with established literature indicating that lentiviral infection engages innate immune sensing pathways (e.g., cGAS-STING or TLR signaling) within DCs, acting as a built-in adjuvant that upregulates co-stimulatory molecules (CD80/CD86) and antigen-presentation machinery (HLA-ABC, HLA-DR) [22,23]. Such maturation is critical for establishing durable immunostimulatory synapses and preventing T-cell anergy, thereby converting DCs into highly immunogenic vehicles [24]. Collectively, our findings indicate that the lentiviral platform not only serves as an antigen delivery vehicle but also actively augments immunogenicity by driving DCs to a state of full functional competence.

The robust T-cell activation induced by Lv-CLDN18.2-DC immunization underscores the efficacy of this strategy. Our data revealed a pronounced CD8+ T-cell-dominant response (Figure 3B). Concomitantly, we observed substantial upregulation of CD25 (Figure 3C), a key activation marker and component of the high-affinity IL-2 receptor, along with elevated secretion of the effector cytokines IFN-γ and TNF-α (Figure 3D). This profile is indicative of a strong Th1-polarized immune response, which is crucial for effective antitumor immunity. Beyond their direct antitumor functions, IFN-γ enhances immunogenicity by upregulating MHC class I expression and promoting immune cell recruitment [25], while TNF-α can mediate direct cytotoxicity against tumor cells and disrupt tumor vasculature [26]. Clinically, a Th1-biased response is associated with favorable outcomes in cancer immunotherapy [27]. The elevated CD25 expression further signifies the generation of highly responsive effector T cells with potent proliferative capacity. Most importantly, these primed CTLs exhibited potent and antigen-specific cytotoxic activity against CLDN18.2-expressing target cells in vitro (Figure 3E). The negligible killing by CTLs primed with the control Lv-Vec-DC confirmed the antigen-dependent specificity of the response, ruling out non-specific effects mediated by the viral vector or allogeneic stimuli.

Our work contributes to and expands the rapidly advancing landscape of CLDN18.2-targeted therapeutics. Chimeric antigen receptor (CAR) T-cell therapy directed against CLDN18.2 has shown promising efficacy in early-phase clinical trials for advanced gastrointestinal cancers; however, it faces challenges including complex manufacturing, high cost, cytokine release syndrome (CRS), and on-target, off-tumor gastric toxicity [28,29]. In parallel, CLDN18.2-specific bispecific T-cell engagers (BiTEs) and antibody–drug conjugates (ADCs) represent potent passive immunotherapeutic modalities currently under intensive investigation [30]. In contrast to these strategies, our DC vaccine-based active immunization approach aims to engage the host’s endogenous immune system to elicit a durable, polyclonal, and self-sustaining response against CLDN18.2. This strategy may offer distinct advantages, including a potentially improved safety profile with a lower risk of severe CRS, and the critical ability to generate long-lived central and effector memory T-cell pools—a foundational immunological advantage of active vaccination over passive antibody transfer or adoptive cell therapies [31,32].

The clinical translation of dendritic cell (DC)-based vaccines is rapidly advancing, reinforcing the translational relevance of our strategy. The regulatory approval of sipuleucel-T for prostate cancer provided a critical proof-of-concept for DC vaccine strategies, demonstrating that ex vivo-activated autologous immune cells can confer clinical benefit [33]. Furthermore, recent advances in personalized DC vaccines loaded with patient-specific neoantigens for melanoma and other solid tumors have shown the capacity to induce de novo, tumor-targeted immunity with measurable therapeutic activity [34]. Our CLDN18.2-targeting DC vaccine directly aligns with this evolving paradigm. By employing a well-defined, frequently expressed tumor-associated antigen (which can serve as a substitute for unique neoantigens), our approach offers a potential alternative to fully personalized neoantigen vaccines. This strategy could significantly reduce manufacturing complexity, production duration, and cost, thereby enhancing its feasibility for broader clinical application and accessibility.

The in vivo experimental data provide strong evidence for the therapeutic potential of our vaccine candidate. In a syngeneic mouse model, administration of three doses of the Lv-CLDN18.2-DC vaccine significantly suppressed tumor growth compared to all control groups (Figure 4C–F). A detailed immunohistochemical analysis of the tumor microenvironment revealed key mechanistic insights. Vaccinated animals exhibited a pronounced increase in tumor-infiltrating CD8^+^ T cells (Figure 4H), directly linking the vaccine-induced systemic immune response to localized antitumor activity. This enhanced immune infiltration correlated with a marked reduction in tumor cell proliferation (as assessed by Ki-67 staining) and, critically, a decrease in the fraction of CLDN18.2-expressing tumor cells. These findings are consistent with the concept of cancer immunoediting, particularly the elimination phase, wherein antigen-specific T cells selectively target and eradicate tumor cells bearing the cognate antigen, thereby applying a selective pressure that can shape tumor immunogenicity [21]. The concomitant decline in Ki-67-positive cells further suggests that the immune response not only eliminates existing tumor cells but also constrains the proliferative capacity of the remaining population, contributing to sustained tumor control.

This study presents a significant advance through a novel dendritic cell (DC) vaccine strategy with several key innovations. First, we targeted CLDN18.2, a clinically validated tumor-associated antigen with a favorable safety profile and high relevance in gastric cancer. Second, we employed a lentiviral vector system that simultaneously enables sustained endogenous antigen expression and promotes potent DC maturation, thereby eliciting a robust and antigen-specific immune response. Collectively, our work provides a comprehensive dataset, from molecular construct design to in vivo therapeutic efficacy, complemented by detailed immunological profiling that delineates the underlying cellular mechanisms.

Our study has certain limitations. The reliance on a single immunocompetent syngeneic mouse model, while valuable, does not fully comprehensively mirror the intricate dynamics of human immune systems and the tumor niche. Future studies should assess the vaccine’s performance across diverse experimental systems, particularly humanized rodent models engrafted with human immune components and hosting CLDN18.2-expressing gastric malignancies, to better predict clinical potential. Furthermore, detailed investigation into the longevity and quality of the induced immunological memory—including the dynamics of memory T-cell subset formation (e.g., central vs. effector memory) and potential epitope spreading—will be crucial for optimizing vaccine efficacy. Given the established synergy of combination therapies in oncology and the pivotal role of the PD-1/PD-L1 axis in tumor-mediated T-cell exhaustion, exploring the combination of our CLDN18.2-targeted DC vaccine (which primes T-cell responses) with PD-1 blockade (which reverses T-cell dysfunction) represents a logical and promising strategy to enhance therapeutic outcomes.

## 5. Conclusions

In conclusion, we have developed and preclinically validated a lentiviral vector-based dendritic cell vaccine targeting CLDN18.2, which elicits potent and antigen-specific antitumor immunity. Supported by the expanding clinical evidence for DC vaccines and the robustness of our data, these findings provide a strong rationale for advancing this immunotherapeutic strategy into clinical trials as a promising treatment for CLDN18.2-positive gastric cancer.

## Data Availability

Data are contained within the article.

## Supplementary Materials

The following supporting information can be downloaded at https://www.mdpi.com/article/10.3390/cancers18030441/s1: Table S1: Sequences of primers for real-time PCR. Figure S1: Generation of stable CLDN18.2-positive gastric cancer cell lines. Figure S2: Analysis of Apoptosis Induced by Different Treatments in AGS~CLDN18.2 and HGC27~CLDN18.2 Cell Lines. Figure S3: Validation of murine bone marrow-derived DC (BMDC) vaccine. Figure S4: Quantitative Analysis of CD25 Expression on CD8⁺ T Cells and Cytokine Levels in Tumor Microenvironment. Original Western blot images.
