# Supplementary material for: Lentiviral Dendritic Cell Vaccine Targeting Claudin-18.2 Elicits Potent Antitumor Immunity Against Gastric Cancer
Source: Cancers (Basel). 2026 Jan 29;18(3):441. doi: 10.3390/cancers18030441 (PMC12897038; doi:10.3390/cancers18030441)

Data Supplementary

Table S1. Sequences of primers for real-time PCR.

Gene	Forward (5'-3')	Reverse (5'-3')
**GAPDH**	GTCTCCTCTGACTTCAACAGCG	ACCACCCTGTTGCTGTAGCCAA
**CLDN18.2**	TTGGGGTTCGTGGTTTCACTG	GTGTACATGTTAGCTGTGGAC

Figure S1: Generation of stable CLDN18.2-positive gastric cancer cell lines.

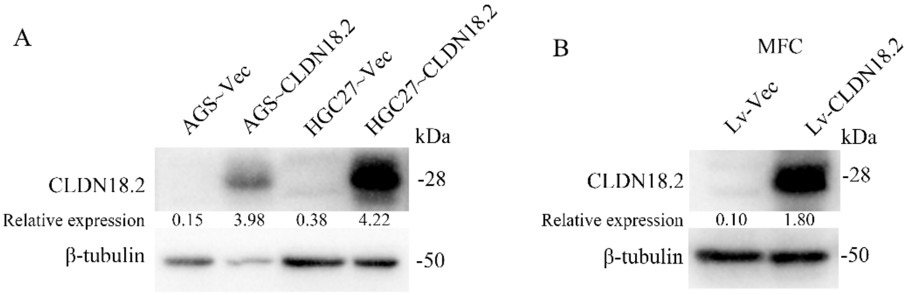

(A) Western blot analysis confirming stable CLDN18.2 expression in human AGS and HGC27 cells after transduction with Lv-CLDN18.2 and puromycin selection. For the original blots, see original blot image 3. (B) Western blot analysis confirming stable CLDN18.2 expression in mouse MFC cells. For the original blots, see original blot image 4.

Figure S2: Analysis of Apoptosis Induced by Different Treatments in AGS~CLDN18.2 and HGC27~CLDN18.2 Cell Lines

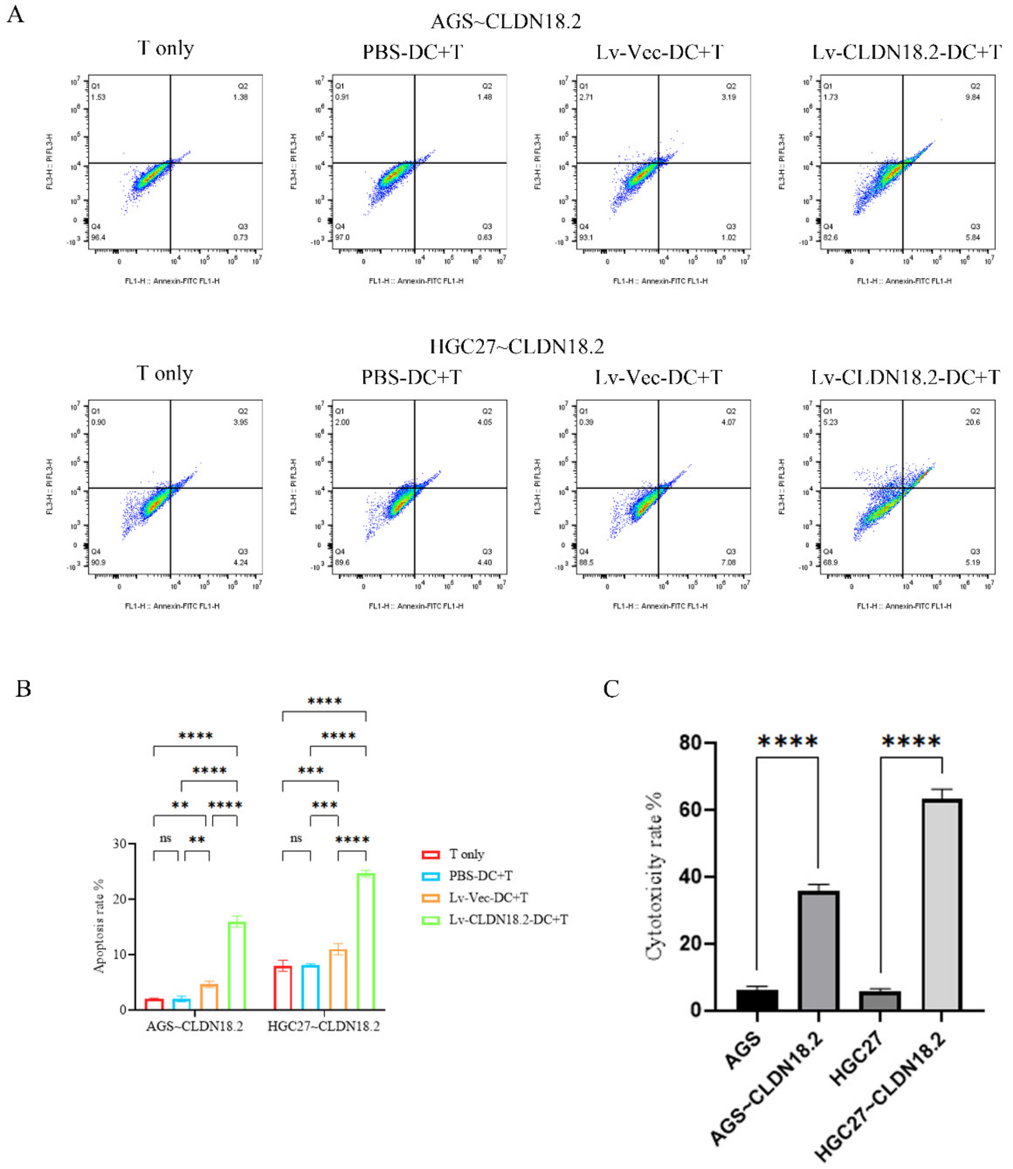

(A) Scatter plots showing the distribution of cells in different quadrants following treatment with T only, PBS-DC+T, Lv-Vec-DC+T, and Lv-CLDN18.2-DC+T in AGS~CLDN18.2 and HGC27~CLDN18.2 cell lines. The plots represent Annexin V-FITC and PI staining, indicating early (Q3) and late (Q2) apoptotic cells. (B) Bar graph depicting the percentage of apoptotic cells in AGS~CLDN18.2 and HGC27~CLDN18.2 cell lines after treatment with T only, PBS-DC+T, Lv-Vec-DC+T, and Lv-CLDN18.2-DC+T. (C) In vitro cytotoxicity assay. Specific lysis of CLDN18.2-negative and CLDN18.2-positive target cells by CTLs at an effector-to-target (E: T) ratio of 40:1. Statistical significance is indicated by asterisks (*$p < 0.05$, **$p < 0.01$, ***$p < 0.001$, ****$p < 0.0001$).

Figure S3: Validation of murine bone marrow-derived DC (BMDC) vaccine.

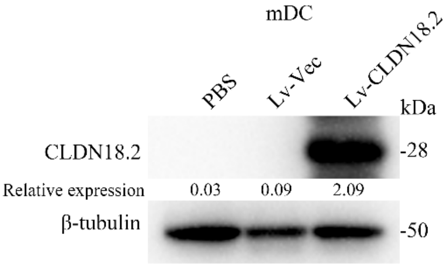

Western blot analysis confirming successful loading of the CLDN18.2 antigen in murine BMDCs transduced with Lv-CLDN18.2. For the original blots, see original blot image 5.

Figure S4: Quantitative Analysis of CD25 Expression on $CD8^+$ T Cells and Cytokine Levels in Tumor Microenvironment

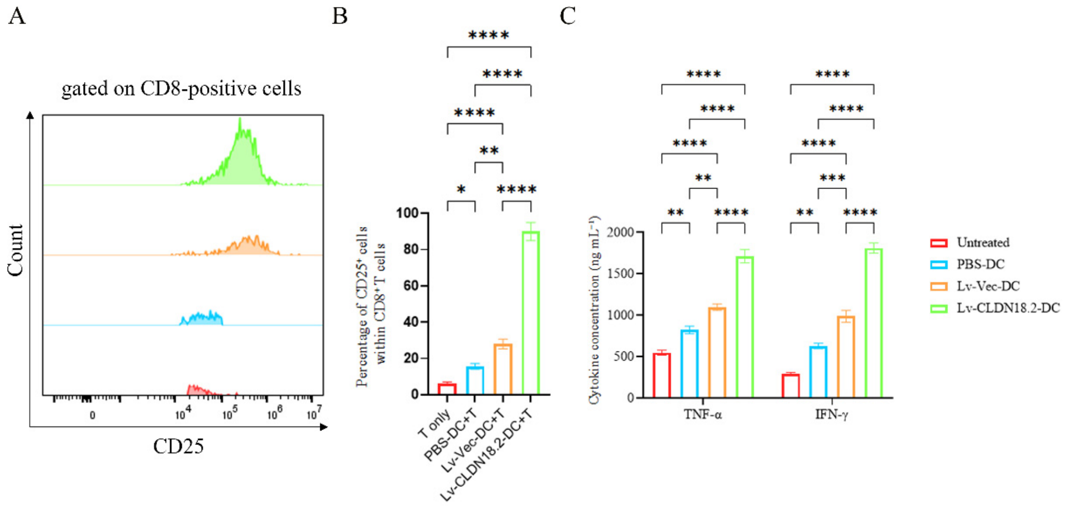

(A-B) Quantitative analysis of CD25 expression on $CD8^+$ T cells in tumors. Data are presented as mean ± SD (n=3). (E) Cytokine levels of IFN-γ and TNF-α in co-culture supernatants, measured by ELISA. Data are presented as mean ± SD (n=3). (C) Bar graph showing TNF-α and IFN-γ concentrations in supernatants from mouse tumor lysates after treatment with untreated, PBS-DC, Lv-Vec-DC, and Lv-CLDN18.2-DC. Error bars represent SEM. Significance levels: *p < 0.05, **p < 0.01, ***p < 0.001, ****p < 0.0001.

Original blot image 1

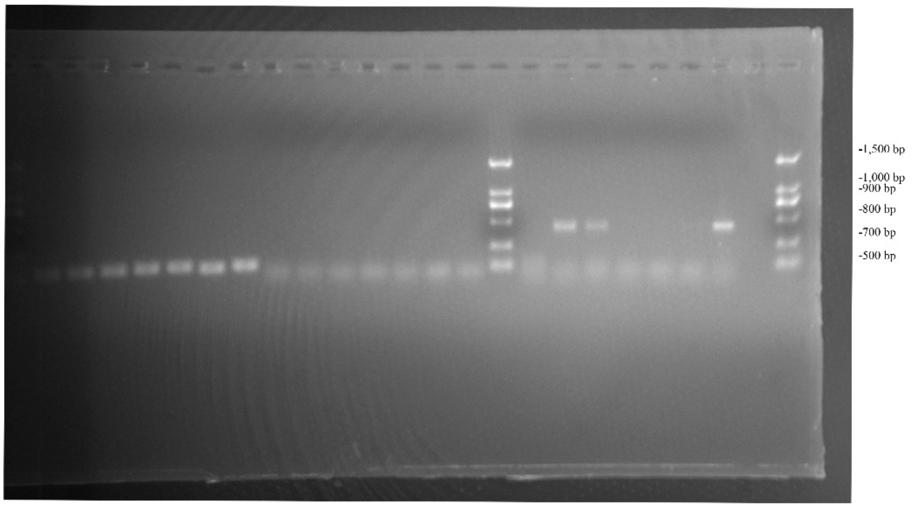

Original blot image 2

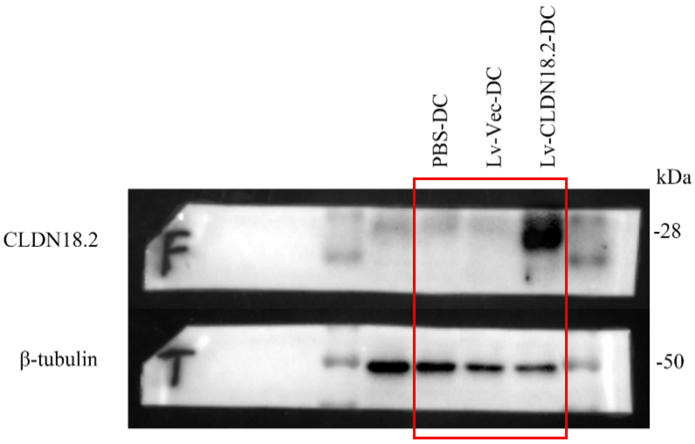

Origina blot image 3

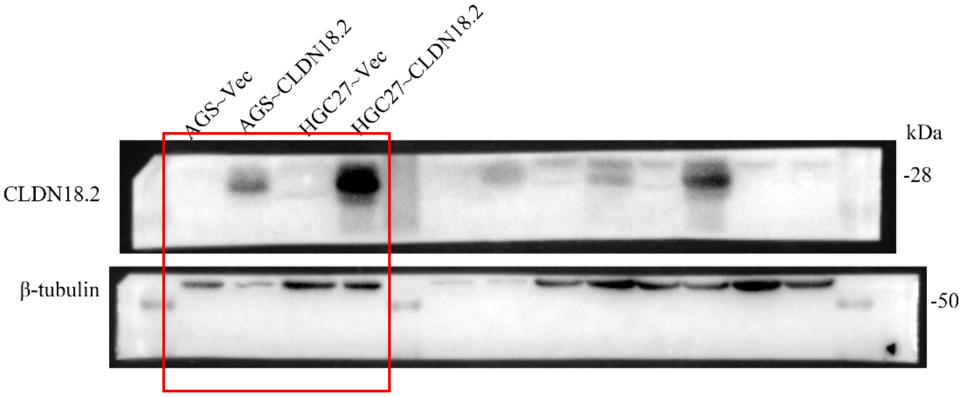

Original blot image 4

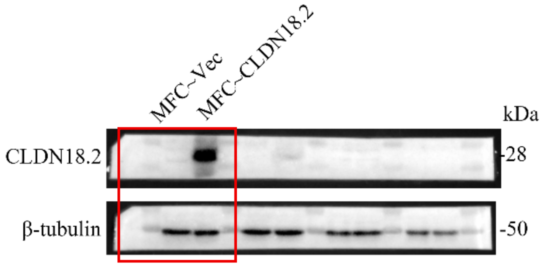

Original blot image 5

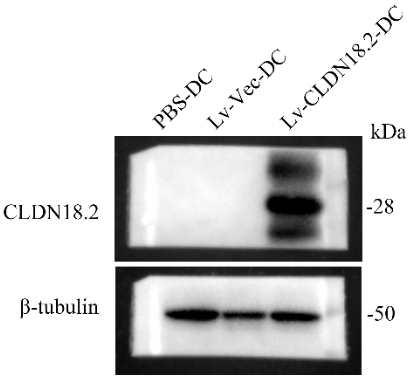

Supplement: Supplementary file 1 [file cancers-18-00441-s001.zip › cancers-4056932-supplementary.pdf]
